# Microbiome–Aging–Wrinkles Axis of Skin: Molecular Insights and Microbial Interventions

**DOI:** 10.3390/ijms262010022

**Published:** 2025-10-15

**Authors:** Varun Challa, Santosh Kumar Prajapati, Surabhi Gangani, Dhananjay Yadav, Lalitha Lekkala, Shalini Jain, Hariom Yadav

**Affiliations:** 1 USF Center for Microbiome Research, Microbiomes Institute, University of South Florida Morsani College of Medicine, Tampa, FL 33613, USA; challa127@usf.edu (V.C.); prajapati11@usf.edu (S.K.P.); dhananjay11@usf.edu (D.Y.);; 2 Center of Excellence in Aging and Brain Repair, Department of Neurosurgery, Brain and Spine, University of South Florida Morsani College of Medicine, Tampa, FL 33613, USA

**Keywords:** microbiome, aging, wrinkle, skin, elastin, MMP

## Abstract

Skin aging is a complex biological process influenced by both intrinsic factors such as hormonal changes, genetic programming, and immunosenescence and extrinsic stressors including ultraviolet (UV) radiation (particularly UV-A and UV-B), pollution, and lifestyle habits. One of the most prominent manifestations of skin aging is wrinkle formation, which arises from the progressive degradation of key extracellular matrix (ECM) components like collagen and elastin. Emerging evidence highlights the skin microbiome as a critical, yet underappreciated, modulator of these structural changes. This review summarizes current understanding of how aging alters skin structure and microbial composition, and how these changes contribute to wrinkle development. Age-associated skin is characterized by reduced hydration, sebum production, and barrier integrity, accompanied by a shift in microbial communities. These microbial shifts promote local inflammation, matrix metalloproteinase (MMP) activation, and oxidative stress, all of which accelerate ECM degradation. We further discuss how commensal microbes and their bioactive products such as probiotics and postbiotics can counteract wrinkle formation. Clinical studies support the efficacy of strains such as *Lactobacillus plantarum* HY7714 and *Bifidobacterium breve* in improving skin elasticity and reducing wrinkle depth. Additionally, this review highlights the emerging role of microbiome-based interventions in skincare, including oral supplements, topical formulations, and postbiotic-enriched products. Overall, we emphasized the therapeutic potential of microbiome modulation as a novel strategy for maintaining skin health and preventing wrinkle formation during aging.

## 1. Introduction

The human skin, encompassing approximately 1.8 square meters, is not only the body’s largest organ but also a complex ecological interface between the host and the environment. Structurally composed of the epidermis, dermis, and subcutaneous tissue, the skin is populated by a diverse community of microorganisms collectively known as the skin microbiome that includes bacteria, fungi, viruses, and microscopic mites [1,2,3]. These microbes inhabit specialized microenvironments across sebaceous, moist, and dry skin regions, with microbial densities reaching up to ten million organisms per square centimeter in some areas [4,5]. The dominant bacterial phyla Actinomycetes, Firmicutes, Proteobacteria, and Bacteroidetes form stable yet site-specific communities that contribute to key physiological functions, including immune regulation, barrier maintenance, and pathogen defense [6,7]. Thus, immune–microbiome crosstalk plays an important role in regulation aging and associated diseases [8].

With aging, the skin undergoes a range of intrinsic and extrinsic changes that alter its structural integrity and function. Intrinsically, aged skin exhibits reduced fibroblast activity, slower cell turnover, decreased hydration, and a thinning dermal matrix. Extrinsically, chronic exposure to ultraviolet (UV) radiation, especially UV-A and UV-B radiation, and environmental pollutants exacerbates oxidative stress and inflammation, accelerating these degenerative changes [9,10,11,12]. Simultaneously, the composition of the skin microbiome shifts with aging and the relative abundance of beneficial strains of *Cutibacterium acnes* decline, while potentially pathogenic taxa such as *Corynebacterium* and *Staphylococcus* increases [13,14]. It is important to note that *Corynebacterium acnes* is a heterogenous species, meaning while some strains offer distinct benefits, others may contribute to increased inflammation [15]. These changes in microbial composition are influenced by reduced sebum secretion, loss of barrier function, and immunosenescence, all of which contribute to microbial dysbiosis and altered host–microbe interactions [16,17].

Emerging evidence suggests that age-related microbial imbalances are not merely reflective of skin aging but may actively participate in the process. The decline in lipophilic commensals and rise in opportunistic bacteria create a pro-inflammatory environment that compromises epidermal homeostasis. This microbial dysregulation has been linked to oxidative stress, matrix metalloproteinase (MMP) activation, and barrier disruption factors that collectively accelerate the onset of visible signs of aging, most notably wrinkle formation [18,19].

Wrinkles represent one of the most recognizable features of aged skin and arise from both intrinsic and extrinsic aging mechanisms. The degradation of collagen and elastin, increased production of MMPs, and the accumulation of advanced glycation end-products (AGEs) contribute to the thinning and weakening of the dermis, leading to sagging and furrowed skin [20,21,22]. Notably, recent studies have identified correlations between microbiome composition and wrinkle severity.

These findings underscore the potential role of the skin microbiome in mitigating age-related dermatological changes. Probiotics such as *Lactobacillus plantarum* and *Bifidobacterium breve* can reduce inflammation, promote ceramide synthesis, and enhance hydration, thereby improving skin elasticity and minimizing wrinkle depth [23,24]. Similarly, postbiotic metabolites like short-chain fatty acids and bacterial lysates can upregulate collagen gene expression, protect against oxidative damage, and restore barrier integrity [25,26]. The role of postbiotics in mitigating inflammation and associated changes in the gut has been noted [27]. Further, clinical evidence further supports that both oral and topical administration of these agents contributes to wrinkle reduction and improved skin texture in older adults. This review addresses the interaction between skin microbiota and host aging mechanisms, highlighting their contribution to wrinkle development. We discuss about how microbial dysbiosis leads to the destruction of collagen and elastin, the extracellular matrix, the barrier, and the process of inflammaging. Mechanistic insights into oxidative stress, i.e., matrix metalloproteinase alteration, are identified as significant contributors to skin aging. Finally, we elaborated the therapeutic strategies, including probiotics, postbiotics, and microbiome-based skincare products, aimed at restoring microbial balance, strengthening the extracellular matrix, and improving skin resilience.

## 2. Microbiome–Skin Interaction During Aging

The structure and functions of the skin change with age due to internal factors such as cellular metabolism, the immune system, and hormonal changes, and external factors such as UV/UV-A/UV-B radiation [10,11,12]. As such, the microbiota can change over the lifetime [28] due to one’s lifestyle and living conditions. [29].

**Figure 1:**
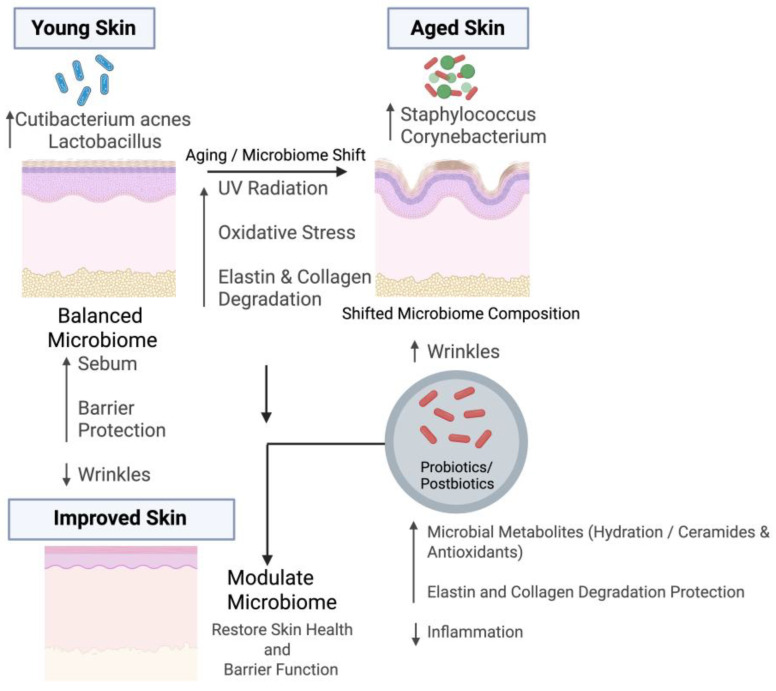
Impact of the Skin Microbiome on Wrinkle Formation and Potential Modulation During Aging. The upper left side represents microbial features of young and healthy skin, which is characterized by higher abundance of *Cutibacterium acnes* and *Lactobacillus* species. These microbes support skin health by producing short-chain fatty acids, maintaining sebum production, and strengthening barrier integrity, which collectively reduce wrinkle formation. In contrast, the upper right side shows aged skin, where an increased abundance of Staphylococcus and Corynebacterium species contributes to inflammation, oxidative stress, and extracellular matrix (ECM) degradation. These changes are driven by aging and environmental factors such as UV exposure. Probiotics and postbiotics can help modulate the aged microbiome by producing beneficial metabolites, enhancing skin hydration, increasing ceramide levels, and suppressing inflammation, ultimately restoring skin health and barrier function. Figure created with BioRender.com.

As one ages, the skin experiences a decrease in sebum and hydration levels. Further, immune dysfunction may occur, which results in significant alterations in skin physiology [9]. As these changes occur, the cutaneous ecology shifts, causing an imbalance in the cutaneous microbiota [16]. Consequently, the skin microbiome differs between younger and older individuals [14]. A report has demonstrated that, in puberty, the density of lipophilic *Cutibacterium acnes* increases along with sebum levels, whereas it is much lower in elderly skin [30]. Furthermore, another report indicated a reduction in Actinomycetes abundance in aged skin [13]. Further, Figure 1 represents the mechanistic insights into the role of the skin microbiome in wrinkle formation and aging.

Therefore, there is a reduction in bacterial diversity during aging. This reduced microbial diversity influence skin physiology by altering lipid metabolism and fostering pro-inflammatory cytokines, thereby contributing to age-related skin dryness and barrier dysfunction [31,32]. It is reported that the diversification and compositional shifts of the skin microbiome in older individuals are closely linked to chronological and physiological aging, driven by impaired barrier integrity, reduced sebum secretion, diminished hydration, and immune senescence [31]. These changes create an unfavorable niche for commensal bacteria while facilitating colonization by opportunistic or pro-inflammatory species [31]. Further, studies have reported that aging skin typically shows a decline in *Cutibacterium acnes*, predominant in youthful sebaceous regions, alongside an increased prevalence of *Corynebacterium* and *Staphylococcus* species, including *S. epidermidis* and the potentially pathogenic *S. aureus* [17,33].

Moreover, systemic aging processes, including immunosenescence and inflammaging, may extend beyond the skin to influence microbial ecosystems at distant sites, such as the gut and oral cavity [19,34]. These interconnected microbiomes—gut, oral, and skin—exhibit correlated aging signatures. In fact, recent machine-learning models have demonstrated that among these, the skin microbiome offers the highest predictive accuracy for chronological age, with deviations within four years on average [35]. This suggests that while microbiome changes may serve as robust biomarkers of aging, their causal role remains debated.

Further research on the skin microbiome has increasingly focused on strategies to restore and maintain its diverse microbial communities. Such approaches may also provide insights into how modulation of the gut microbiota in older individuals could prevent or mitigate age-related skin dysfunction [33]. For example, Dimarzio et al. demostrate that the stratum corneum can experience enhanced ceramide levels when *Streptococcus thermophilus* is topically applied on the skin [36]. Recently, oral and topical probiotics have been proposed as a therapy to restore microbiota balance, support skin barrier function, and protect against environmental factors, especially UV-B induced skin damage [37,38]. Huang et al. implicate that UV-A-induced photoaging impairs autophagic degradation in dermal fibroblasts by reducing lysosomal acidification and cathepsin activity, heavily influencing skin photoaging [39]. Interestingly, probiotics may help restore the balance between free radical removal and production, which may slow aging [40]. Oral and topical compounds are being explored for their potential to modulate the skin microbiome [41]. *Orobanche rapum* extract promotes skin rejuvenation and protects the cutaneous microbiota, promoting healthier skin [42]. Recently, the term photobiomics has been introduced to describe the use of low levels of visible or near-infrared light to influence the gut microbiome through photo-biomodulation [43].

Overall, aging-driven changes in skin physiology disrupt the microbial balance, reducing commensals and favoring opportunistic species. Probiotic and postbiotic interventions may reveal a promising approach to restore skin homeostasis. These insights implicate understanding how microbiome alterations directly contribute to wrinkle formation (Table 1).

## 3. Microbiome Associated with Wrinkles

The relationship between skin microbiome composition and skin aging is complex and multifaceted. A diverse and balanced microbiome is generally associated with healthy and young skin [18]. Skin microbial communities influence skin aging by regulating immune responses, protection against UV damage, maintenance of skin barrier function, production of beneficial metabolites, and modulation of skin pH. Research has identified several microbial pathways that may be particularly relevant to skin aging, such as the biosynthesis of antibiotics and other metabolites that can influence skin health [13,18]. Specific commensal bacteria produce AMPs and other products that inhibit the growth of pathogens. This protects skin barrier function and reduces inflammation, which indirectly slows wrinkle formation.

Certain bacterial species may be associated with increased or decreased wrinkle formation. Bacteria associated with reduced wrinkle formation include certain species of *Cutibacterium acnes* and *Lactobacillus*. *Cutibacterium acnes* is typically dominant in younger skin and has been linked to reduced signs of aging. This species is known to play a role in maintaining skin health by producing short-chain fatty acids and other metabolites that help maintain skin barrier function [18,19]. Its abundance tends to decrease with age, particularly in individuals over 55–60 years old. While not typically abundant on the skin, some *Lactobacillus* species have been associated with anti-aging effects. In contrast, some bacteria such as *Corynebacterium* and *Staphylococcus* species have been associated with increased wrinkle formation. In this context, Blaise et al. demonstrate that the increased abundance of *Corynebacterium* species is responsible for producing keratolytic effect [48]. This shift may be related to changes in skin physiology that occur with age. While some *Staphylococcus* species are common skin commensals, an overabundance of certain strains has been associated with skin aging and inflammation. The balance of different *Staphylococcus* species may play a role in skin health and overall appearance [18,19].

In the therapeutic perspective by Lee et al., it was demonstrated that the oral administration of *Lactobacillus* strains, such as *L. plantarum* HY7714, may improve skin hydration and reduce wrinkle depth [23]. Similarly, Bouilly-Gauthier et al. demonstrate that oral consumption of a synbiotic formulation comprising *Lactobacillus johnsonii* and carotenoids improves skin resistance to high UV-A exposure and natural sunlight [49]. Furthermore, Rong et al. implicate that *Lactobacillus helveticus* supernatant increases resistance to UV-B-induced oxidative stress and hyperpigmentation [50]. As our understanding of the skin microbiome’s role in aging continues to evolve, there is a growing interest in developing microbiome-based interventions to promote skin health and reduce signs of aging (See Table 2).

## 4. Understanding of Microbiome on Wrinkle Formation During Aging

**Figure 2:**
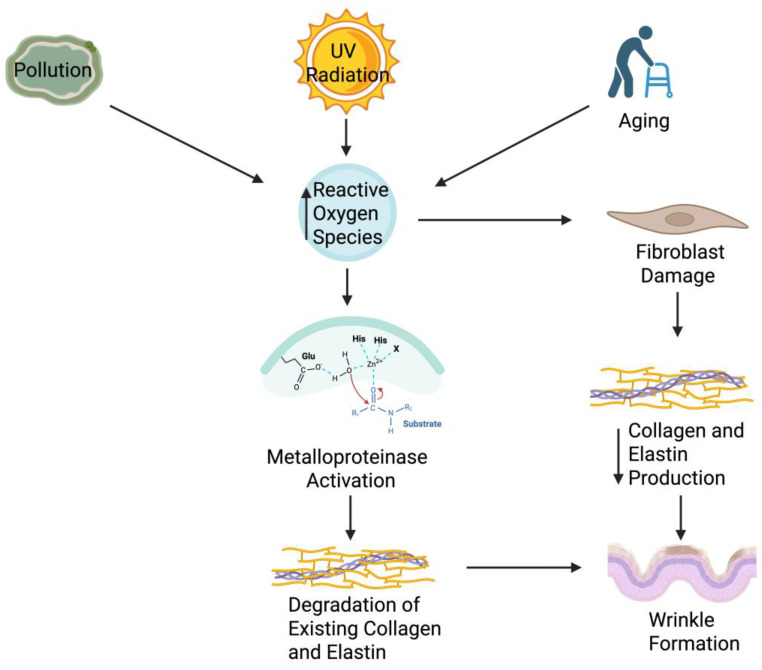
Mechanisms of Wrinkle Formation via Extracellular Matrix Degradation. Extrinsic stressors like pollution and UV radiation, together with intrinsic aging, elevate reactive oxygen species (ROS). ROS activate MMPs, which degrade dermal collagen and elastin, while fibroblast damage limits ECM synthesis. These changes weaken skin structure and promote wrinkle formation. The figure highlights MMP activation, underscoring oxidative stress as a key driver of ECM breakdown. Figure created with BioRender.com.

The skin microbiome plays a crucial role in wrinkle formation by modulating extracellular matrix integrity through immune and oxidative pathways. Beneficial microbes help maintain collagen and elastin stability by reducing inflammation and oxidative stress, whereas dysbiosis promotes MMP activation, collagen degradation, and accelerated wrinkle development (shown in Figure 2).

### 4.1. Mechanistic Overview of Wrinkle Formation in Aging Skin

As people age, their skin undergoes physiological changes that affect its structure and composition. It has been demonstrated that reduced elastin and collagen synthesis is one of the factors responsible for wrinkle formation [51]. As a result, the dermis thins, leading to sagging and deeper lines. In addition, a report has shown that chronic exposure to UV-B further accelerates wrinkle development [52,53]. Evidence indicates that wound healing capacity declines with age as well, largely due to reduced fibroblast and keratinocyte activity, along with a diminished inflammatory response [53,54]. Moreover, Lähteenvuo et al. implicate that a reduction in angiogenesis in older individuals leads to reduced amounts of oxygen and nutrients available for the tissues, hindering the healing process as well [55]. Another study, Berard et al., shows that aging correlates with a reduction in immunological responsiveness, leading to a diminished cutaneous reactivity to allergens [56]. At the same time, deterioration of the skin barrier permits greater penetration of irritants and pathogens, thereby increasing susceptibility to infections and further highlighting the detrimental effects of aging on skin health [56]. Therefore, there is a clear need to further explore this area to better understand the mechanisms underlying age-related skin vulnerability and to develop strategies that can strengthen barrier function and enhance cutaneous immunity.

Skin aging is a complex, multifactorial process involving both intrinsic (genetic) and extrinsic (environmental) factors. Intrinsic factors include turnover, diminished moisture content, and thinning of the dermis. Extrinsic aging, primarily driven by UV radiation (photoaging), pollution, and lifestyle factors like smoking, exacerbates these changes [57].

Alkawar et al. have shown that UV-B exposure induces p53 target genes *p21* and DNA polymerase eta (*pol η*) in keratinocytes and skin explants, but this response was diminished when IGF-1 signaling was inhibited [58]. Since *p21* and pol *η* help prevent mutagenic DNA replication, reduced IGF-1 activity in aged skin may impair p53 function, thereby increasing susceptibility to nonmelanoma skin cancers in older individuals [58].

Wrinkles, both fine lines and deeper furrows, form because of these changes. They are most observed in sun-exposed areas, reflecting the chronic impact of environmental stressors. At the molecular level, chronic UV-B exposure stimulates the overproduction of ROS, which in turn activate MMPs [59]. MMPs degrade critical structural proteins such as collagen, elastin, and fibronectin, leading to reduced dermal integrity and elasticity [60]. Additionally, repeated facial expressions contribute to dynamic wrinkle formation, while intrinsic aging leads to static wrinkles due to progressive collagen loss and fragmentation (Figure 2). Together, these processes highlight that wrinkles are not merely superficial cosmetic concerns but rather a visible outcome of complex biological events that compromise the structural integrity of the skin.

### 4.2. Elastin Degradation and Microbiome Interactions

Elastin is a highly resilient protein, forming a network in the skin’s dermis that allows tissues to stretch and recoil. It is produced by fibroblasts during early development and remains relatively stable throughout life, but its synthesis declines after maturity [20]. With aging, elastin fibers become fragmented and cross-linked due to enzymatic degradation (mediated by enzymes like elastase) and the accumulation of AGEs. This leads to a loss of skin elasticity, contributing to the appearance of sagging and wrinkles [61]. Solar elastosis, a hallmark of photoaged skin, occurs when prolonged UV exposure damages elastin fibers. The result is the accumulation of abnormal, disorganized elastin in the skin. Intrinsically, as we age, the skin undergoes structural and functional changes, including thinning of the dermis, which further weakens the skin’s elasticity and increases the prominence of wrinkles [62].

Emerging research indicates that the skin microbiome also influences elastin homeostasis. As indicated by Cheung et al., certain pro-inflammatory strains of *Cutibacterium acnes* and *Staphylococcus epidermidis* produce enzymes like proteases and elastases that can degrade the components of the extracellular matrix [63]. Conversely, Shirzad et al. explain commensals like *Lactobacillus* species may help to preserve the elastin integrity by inhibiting elastase activity [64]. An imbalance in the skin microbiome can thus accelerate elastin degradation, contributing to wrinkle formation. Moreover, UV radiation promotes the production of ROS, which upregulate MMPs and elastases, accelerating elastin degradation. This not only replaces healthy elastin fibers but also disrupts the surrounding ECM, further diminishing skin elasticity and contributing to coarse wrinkling and uneven skin texture [22]. UV-A radiation is a well-known inducer of ROS that can alter elastic fiber structure, thereby reducing skin elasticity and contributing to wrinkle formation and aging [11,12]. At the molecular level, the degradation of elastin reflects the interplay between intrinsic genetic programming and extrinsic environmental insults. Aging skin also experiences reduced fibroblast activity and dermal thinning, which together limit the skin’s capacity to repair and regenerate its elastic fiber network [65]. Clinical and experimental evidence indicates that wrinkle formation is significantly associated with UV-B-induced reduction in skin elasticity and structural modifications of elastic fibers. UV-B activates keratinocyte-derived cytokines that increase fibroblast elastase activity, which breaks down elastic fibers [53]. Elastase inhibition is crucial in preventing wrinkle formation, highlighting fibroblast elastase as a key mediator in the mechanism of UVB-induced wrinkle formation [53].

Therefore, elastin serves as a cornerstone of dermal structure and function. Its gradual degradation through enzymatic activity, oxidative stress, and glycation, compounded by external factors such as UV-A/UV-B exposure, is central to the pathophysiology of skin aging [66]. Understanding these mechanisms highlights why strategies targeting oxidative stress, glycation, and enzymatic degradation are key to preventing and reducing wrinkles and maintaining youthful skin integrity.

### 4.3. Role of MMP-1 and MMP-9 in Wrinkle Formation

MMPs, particularly MMP-1 and MMP-9, are key enzymes involved in the degradation of extracellular matrix components during skin aging and can lead to increased wrinkle formation. MMP-1 breaks down fibrillar collagen types I and III [67]. On the other hand, MMP-9 contributes to gelatin and elastin degradation [68]. These enzymes are upregulated in response to ultraviolet radiation, oxidative stress, and chronic inflammation. This is achieved largely through the activation of the AP-1 and NF- κB signaling pathways [69]. This enzymatic activity leads to collagen fragmentation, dermal thinning, and wrinkle formation. In UV-B-induced photoaging, elevated MMP-1 and MMP-9 expressions are correlated with collagen degradation and visible wrinkling. Further, the skin microbiome has been identified as a key environmental modulator of MMP activity [70]. Further, Tirka et al. demonstrate that elevated serum MMP-1 levels are significantly associated with increased severity of facial wrinkles in photoaging [71]. A moderate positive correlation was observed between MMP-1 levels and wrinkle scores, with nearly half of the variance in wrinkle severity explained by MMP-1 expression [71]. Moreover, Hong et al. show that lipoteichoic acid (pLTA) from Lactobacillus plantarum inhibits UV-induced MMP-1 expression, suppresses MAPK/AP-1 and NF-κB activation, reduces ROS generation, and enhances type I procollagen synthesis in human dermal fibroblasts [72]. These findings suggest that pLTA may serve as a promising agent for the prevention and treatment of skin photoaging [72]. Oral administration of *Lactobacillus plantarum* HY7714 significantly suppressed MMP expression in experimental models, thus preserving collagen levels and reducing wrinkle depth [73]. These results were supported in a clinical trial where *Lactobacillus plantarum* HY7714 supplementation improved both skin elasticity and reduced trans epidermal water loss in human subjects [23]. Both probiotics and postbiotics have been shown to inhibit MMP activity indirectly. The suppression of MMPs by these probiotic strains is believed to occur through the downregulation of oxidative stress and pro-inflammatory cytokines that are upstream activators of MMP expression. This highlights the microbiome’s potential as a regulator of host signaling pathways such as NF-κB and AP-1, both of which are involved in MMP-9 upregulation [74]. This suggests that targeted microbial interventions may help suppress MMP-mediated matrix degradation and slow wrinkle formation.

### 4.4. Collagen and ECM Breakdown in Skin Aging: Microbial and Molecular Modulation

In addition to elastin, collagen is a critical structural protein in the dermis that provides tensile strength to the skin [75]. Type I and III collagens are predominant in youthful skin, forming tightly packed fibers that maintain the skin’s integrity. Type I collagen is the most abundant and primarily contributes to resistance to mechanical forces, while Type III collagen supports skin elasticity and is more prevalent during early wound healing [76]. During aging, collagen production decreases while its degradation increases [77]. This is primarily due to the upregulation of MMPs triggered by prolonged UV exposure, oxidative stress, and inflammation. An in vitro study shows that UV exposure increases MMP-1 expression in dermal fibroblasts, which degrades type I collagen [69]. It has been demonstrated that UV-B exposure in specific increased MMP-1 and MMP-9 expression, leading to the degradation of type I collagen and dermal thinning [23]. As the collagen breaks down, the thickness of the skin is consequently reduced, causing the formation of wrinkles.

In this context, emerging evidence indicates that the skin microbiome can also modulate collagen turnover, positioning it as a potential upstream regulator of age-related changes in dermal structure [78].

The skin harbors a complex ecosystem of commensal and pathogenic microbes that interact closely with host immune and structural systems. Specific commensals like *Lactobacillus plantarum* and *Streptococcus thermophiles* have been shown to preserve collagen integrity by reducing inflammation and oxidative stress, which leads to decreased metalloproteinase activity [79]. These beneficial strains may also influence host gene expression and barrier function, further stabilizing the extracellular matrix. Conversely, the overgrowth of pro-inflammatory strains of *Cutibacterium acnes* or loss of microbial diversity can exacerbate collagen degradation through the production of collagenolytic enzymes [80]. This dysbiosis may act synergistically with external stressors like UV exposure, accelerating the aging process by weakening the skin’s structural integrity.

Other extracellular matrix components such as fibronectin and hyaluronic acid play essential roles in maintaining dermal structure and hydration. Fibronectin is involved in both wound healing and cell adhesion. Its reduction contributes to decreased skin regeneration [81]. Hyaluronic acid, on the other hand, retains water and helps keep the skin plump and elastic. As one ages, hyaluronic acid production declines, contributing to skin dryness and fine lines [82]. The deterioration of these extracellular matrix elements contributes to the loss of dermal volume and elasticity, increasing wrinkle depth and skin looseness (Table 3).

A recent study indicates that microbial imbalances contribute to the breakdown of the extracellular matrix by promoting inflammation and oxidative stress [83]. Another study implicates blocking TNFα with etanercept reduced UV-B-induced recruitment of inflammatory cells and inhibited MMP13 expression. However, it also decreased mature collagen, increased collagen fragmentation, and lowered procollagen levels. These results suggest that while TNFα blockade limits inflammation, it may impair collagen synthesis and matrix integrity in UV-B-irradiated skin [84]. Importantly, UV-A irradiation accelerates photoaging by inducing oxidative stress, DNA damage, collagen degradation, and cellular senescence in dermal fibroblasts [85]. This study demonstrates that rapamycin counteracts these effects by enhancing autophagy, reducing p53 and phosphorylated HSP27 levels, limiting oxidative and genotoxic stress, and preserving collagen via activation of the TGF-β/Smad and MAPK/AP-1 pathways [85]. These findings underscore the potential of probiotic and postbiotic intervention in restoring the microbial balance and mitigating age-related structural damage.

**Table 3:** Mechanism of Wrinkles and the Role of the Microbiome.

Wrinkle Formation Contributor	Mechanism of Action	Microbiome Influence
Collagen Degradation	Matrix metalloproteinases break down collagen, and are activated by factors such as UV radiation, reactive oxygen factors, and inflammation.	The microbiome modulates inflammation. Good microbiota help suppress inflammation, keeping matrix metalloproteinases levels low. Certain microbes also produce antioxidants that neutralize oxidants [86]
Oxidative Stress	Reactive oxygen species cause wrinkles by damaging the dermal fibroblasts, activating matrix metalloproteinases, and leading to a reduction in skin elasticity.	Certain skin bacteria produce antioxidant enzymes that neutralize ROS [87]
Inflammation	Inflammation activates matrix metalloproteinases, damage the fibroblasts that produce collagen, and increase overall oxidative stress.	Healthy skin microbiota help suppress inflammation by inhibiting immune pathways and creating anti-inflammatory cytokines [88]
Loss of Skin Hydration	The stratum corneum contains high levels of water, maintaining the elasticity of the skin.	Commensal bacteria promote tight junction integrity and lipid production, which leads to less trans epidermal water loss and better moisture retention, keeping the skin elastic [89]

## 5. Therapeutic Approach

### 5.1. Probiotics/Postbiotics in Reducing Wrinkles

**Figure 3:**
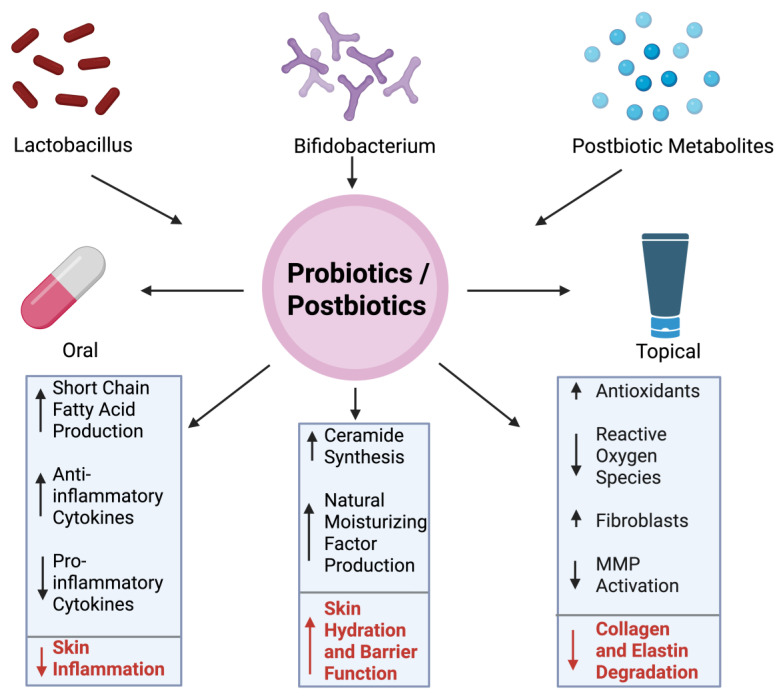
Mechanism of Wrinkle Reduction via Probiotics/Postbiotics. The central circle represents probiotic and postbiotic interventions, which can be administered either orally or topically. Benefits of these two include increased production of short-chain fatty acids, modulation of cytokine profiles to reduce inflammation, promotion of ceramide synthesis and natural moisturizing factor production, antioxidant generation, suppression of ROS, reduced MMP activation, and preservation of fibroblasts. Together, these mechanisms help protect collagen and elastin from degradation and contribute to healthier, more youthful skin. Figure created with BioRender.com.

The role of probiotics and postbiotics in reducing wrinkles has become increasingly popular, highlighting the importance of the gut-skin axis. Probiotics are live microorganisms, while postbiotics are the byproducts or metabolites produced by these microbes. As the skin ages, there is less production of elastin and collagen, as well as diminished hydration and increased oxidative stress [90]. All these factors can contribute to the formation of wrinkles. Probiotics can influence these processes in multiple ways. For instance, probiotic strains like *Lactobacillus* and *Bifidobacterium* species have been shown to reduce markers of inflammation and suppress the production of inflammatory cytokines, thereby limiting collagen breakdown and preserving the integrity of the skin [91]. Topical applications directly interact with the epidermal layer, which promotes resistance to the effects of aging on skin. Postbiotics like short-chain fatty acids and lipoteichoic acids have antioxidant effects, relieving the stress caused my environmental stressors such as UV radiation and pollution [92]. Postbiotics have also been found to increase skin hydration by stimulating the production of ceramides and natural moisturizing factors, which decreases the presence of fine lines on the skin [93]. Some fermented skincare products, which contain postbiotics, can upregulate the expression of genes involved in collagen synthesis, supporting the dermal integrity and minimizing the production of wrinkles as well [94]. Probiotics may also reinforce the skin barrier and prevent transdermal water loss, keeping the skin hydrated and healthy (Figure 3).

Figure 3 represents the effect of probiotics and postbiotics on skin.

Probiotics, particularly strains such as *Lactobacillus* and *Bifidobacterium*, are recognized for their ability to modulate skin health through both the gut-skin and skin-skin axes. These microbes can be administered orally or applied topically, with each route offering distinct mechanisms for improving skin condition. Probiotics that are taken orally help to restore the gut microbiome, which can reduce systemic inflammation and oxidative stress, both of which contribute to dermal aging [95]. As highlighted in a study, strains like *L. plantarum* and *B. breve* have demonstrated the ability to boost anti-inflammatory cytokines such as IL-10 while downregulating pro-inflammatory markers like IL-6, leading to lower skin inflammation and improved elasticity [73]. Topical probiotics enhance the skin barrier and microbial diversity by promoting the colonization of beneficial microbes. This acts to outcompete harmful strains associated with aging [96].

Probiotics can combat wrinkles by influencing processes such as skin hydration and collagen stability [23]. Furthermore, probiotics have been linked to increased production of ceramides and NMFs, both of which help prevent water loss in the skin. This helps the skin remain moisturized and plump. In addition, probiotics indirectly inhibit MMPs enzymes that degrade collagen and elastin by reducing inflammation and oxidative stress [24]. By preserving the extracellular matrix’s integrity, the formation and appearance of fine lines and wrinkles is reduced. Probiotics also support fibroblast viability, which helps maintain dermal structure and repair UV-induced damage [97]. Mitochondrial dysfunction in aging fibroblasts leads to excess ROS production, which triggers DNA damage responses and promotes cellular senescence. As a result, collagen synthesis is reduced while the secretion of matrix-degrading enzymes is increased, leading to an accelerated rate of dermal aging [98].

Postbiotics, which include short-chain fatty acids (SCFAs), lipoteichoic acids, and bacterial peptides, are the products of probiotics [25]. SCFAs such as butyrate exhibit anti-inflammatory and antioxidant effects, which help to neutralize ROS that accumulate from stressors such as UV radiation or pollution. These metabolites also signal the upregulation of genes involved in ceramide synthesis and skin barrier repair. Moreover, fermented skin care products rich in probiotics have been found to directly stimulate collagen gene expression, improving dermal density and smoothness [26] (Figure 3). Apart from probiotics and postbiotics, natural products like fish oils rich in docosahexaenoic acid (DHA) show effectiveness against aging-induced skin oxidation.

DHA is readily incorporated into fibroblast membrane phospholipids, and UVA irradiation promotes the generation of oxidized DHA-derived lipids that activate Nrf2 signaling [99]. In DHA-supplemented cells, Nrf2 enhances protective gene expression, whereas Nrf2-deficient fibroblasts show increased TNFα and MMP13 induction along with higher levels of oxidized phospholipids. These findings suggest that an intact Nrf2 pathway is essential to counteract DHA-induced inflammation and matrix degradation under UV stress [99]. Further, *Alchemilla* and *Chamomile* represent rich sources of bioactive compounds with well-documented dermatological benefits, ranging from anti-inflammatory, antioxidant, and antimicrobial effects to anti-aging and regenerative activities [100,101]. Their traditional use is now substantiated by modern research, positioning them as promising candidates for cosmetic and therapeutic applications, while future studies should emphasize clinical validation and optimized formulations.

### 5.2. Clinical Evidence and Preventive Potential of Probiotics and Postbiotics in Skin Aging

Clinical trials have yielded results that indicate the potential probiotics and postbiotics have in preventing wrinkles. Oral supplementation of *Lactobacillus plantarum* HY7714 was associated with improved skin elasticity and reduced wrinkle depth after several weeks of use [23]. Similarly, postbiotic-containing creams have been shown to reduce wrinkle severity and increase the dermal density in both human and animal subjects [93]. All these studies indicate that an effective strategy for preserving the skin can be to address the skin’s microbiome. Each probiotic and postbiotic can have variable effects and address different aspects of the skin, highlighting the importance of continued research on the skin microbiome. In total, probiotics and postbiotics have demonstrated the capability to reduce the appearance of wrinkles through various pathways, such as enhancing skin barrier function of the skin, reducing inflammation, increasing hydration, or stimulating the production of certain useful proteins. Further, there are some marketed products highlighted in Table 4.

## 6. Diagnostic Potential of Skin Microbiome Profiling

Recent technological advances have led to the development of non-invasive diagnostic approaches using skin microbiome signatures to assess current and future skin conditions. Multi-omics and machine learning models have demonstrated that the skin microbiome can serve as a reliable biomarker for chronological and biological aging [35]. Similarly, large-scale metagenomic and imaging-based surveys have enabled the formulation of indices such as the Facial Aging Index, which integrates skin physio-optical parameters with microbial composition to assess aging-related changes [45]. Moreover, 16S rRNA and shotgun metagenomic analyses are increasingly being utilized in dermatological diagnostics to detect microbial dysbiosis linked to conditions such as acne, eczema, rosacea, and atopic dermatitis—conditions that often precede or accelerate skin aging [44,46].

## 7. Conclusions

The skin microbiome plays an essential role in the aging process and maintenance of skin health. Age-related shifts disrupt barrier integrity, create more inflammation, and accelerate the process of collagen and elastin breakdown, all of which contribute to the formation of wrinkles. Probiotics containing *Bifidobacterium* and *lactobacillus* and their lysate as a postbiotic can restore microbial balance and prevent inflammation, keeping the skin well-kept. Early clinical trials demonstrate that improvements in skin elasticity, hydration, and wrinkle depth, highlighting the importance of microbiome-targeted approaches. Further clinical trials and mechanistic studies will be essential in translating these findings into anti-aging solutions.

## Figures and Tables

**Table 1:** Role of Microbiome on Aging Skin.

Study	Changes	Mechanism
Randomized, double blind, placebo-controlled trial	Decreased transepidermal water loss and wrinkle depth, with an increase in skin gloss and skin elasticity.	HY7714 modulates the expression of enzymes involved in ceramide synthesis and protects against ultraviolet radiation. HY7714 also inhibits the activity of metalloproteinases, which degrade collagen and elastin in the skin [44].
Multi Center and Deep Sequencing Survey	Aging, skin physio-optical conditions, and facial microbiome.	Aging influences skin microbiomes. The Facial Aging Index can see changes in the skin microbiome to assess aging [45].
Observational Study	Significant changes were observed in aging at three body sites examined (face, buttocks, and arm). Decreased sebum and increased lipids/natural moisturizing factors (NMFs)/antimicrobial peptides (AMPs).	Aging reduces the amount of sebum through hormonal changes and alterations in the sebaceous glands. Changes in the lipids or NMFs and AMPs cause changes in the skin microbiome [46].
Cross-sectional, observational microbiome	Significant decrease in Actinomycetes and an increase in *Corynebacterium kroppenstedtii* in the older group (>55 years old). Significantly higher proportion of *Cutibacterium acnes* and *Lactobacillus crispatus* in the younger group (18–35 years old).	Younger skin produces more sebum, creating an oily environment that promotes the development of bacteria like *Lactobacillus crispatus*. Older skin has reduced sebum production, leading to the promotion of the development of *Corynebacterium kroppenstedtii* [47].

**Table 2:** Studies evaluating skin microbiome and wrinkle-associated outcomes.

Study/Product	Model/Population	Microbial Strain/Component	Mechanism	Observed Effects on Skin/Wrinkles
Blaise et al.	Human, aged skin	*Corynebacterium* spp.	Skin microbiome profiling	Increased keratolytic activity; associated with wrinkle formation [48].
Lee et al.	Clinical (oral supplementation)	*Lactobacillus plantarum* HY7714	Oral probiotic	Improved skin hydration and reduced wrinkle depth [23].
Bouilly-Gauthier et al.	Human, clinical trial	*Lactobacillus johnsonii* + carotenoids (synbiotic)	Oral	Increased resistance to UV-A and sunlight-induced damage [49].
Rong et al.	In vitro/animal model	*Lactobacillus helveticus* supernatant	Topical/supernatant exposure	Reduced UVB-induced oxidative stress and pigmentation [50].

**Table 4:** Microbiome- and Synbiotic-Based Products for Skin Health and Anti-Photoaging Applications.

Product Type	Name of Product	Content	Mechanism
Topical Serum	Gallinée Youthful Serum	Prebiotics, probiotics, and postbiotics	Improves the skin barrier, hydrates the skin, and reduces overall inflammation, all of which lead to healthier and younger looking skin. [102]
Oral capsule	Pendulum Skin probiotic	Probiotics strains (*Akkermansia muciniphila* and *Clostridium butyricum*) and prebiotics (*Chicory insulin*)	Supports gut microbiome balance, reducing inflammation and stress, leading to healthier skin. [103]
Topical Cream	Aurelia London probiotic B-Hydrated Moisturizer	Probiotic strains (*Bifidobacterium*) and prebiotics (inulin)	Suppress the skin immune response, preventing inflammation. Moisturizes the skin. https://www.aurelialondon.com/collections/moisturiser (accessed on 28 March 2025)
Topical Serum	Esse Probiotic Serum	Probiotics (*Lactobacillus* and *Bifidobacterium*) and prebiotics (inulin)	Probiotics outcompete harmful bacteria, stimulate collagen production, all of which increase the strength of the skin barrier. https://us.esseskincare.com/product/probiotic-serum/?srsltid=AfmBOopJFB37shr1IpubtR5yi92y2ZN-uLqk0k2_dXz0J7L8h8aXbfiX (accessed on 28 March 2025)
Topical Skincare	Lavera Barrier Balance Skincare Range	Prebiotics (Inulin and *Lactobacillus* ferment)	Nourish the beneficial skin bacteria, strengthening the skin’s natural barrier. https://www.lavera.com/products/care-series/barrier-balance-skin-care-series (accessed on 28 March 2025)
Oral Synbiotic Supplement	Patients with mild atopic dermatitis	*Bifidobacterium animalis* subsp., *Lactis BS01*, *Lactiplantibacillus Plantarum LP-14* and *Lacticaesibacillus Rhamnosus LR05* with *Fructooligosaccharides* and *riboflavin*	Significant improvements in itching and redness as well as a reduction in lesion severity. https://www.nutraingredients-usa.com/Article/2024/04/25/Probiotical-study-links-synbiotic-with-improvements-in-skin-conditions/ (accessed on 28 March 2025)
Oral Synbiotic Supplement	Adults with melasma	*Lactococcus lactis*, *Lactobacillus acidophilus*, *Lactobacillus casei*, *Bifidobacterium longum*, *Bifidobacterium infantis*, *Bifidobacterium bifidum*	https://onlinelibrary.wiley.com/doi/10.1111/jocd.13955? (accessed on 28 March 2025)
